# Epigenetic evidence for involvement of the oxytocin receptor gene in obsessive–compulsive disorder

**DOI:** 10.1186/s12868-016-0313-4

**Published:** 2016-11-30

**Authors:** Carolina Cappi, Juliana Belo Diniz, Guaraci L. Requena, Tiaya Lourenço, Bianca Cristina Garcia Lisboa, Marcelo Camargo Batistuzzo, Andrea H. Marques, Marcelo Q. Hoexter, Carlos A. Pereira, Euripedes Constantino Miguel, Helena Brentani

**Affiliations:** 1 Department of Psychiatry, School of Medicine, University of São Paulo, R. Dr. Ovídio Pires de Campos, 785, 3º andar, sala 9, São Paulo, SP 05403-010 Brazil; 2 Institute of Mathematics and Statistics, University of São Paulo, São Paulo, Brazil

**Keywords:** OCD, Methylation, Oxytocin receptor gene (*OXTR*)

## Abstract

**Background:**

Obsessive–compulsive disorder (OCD) is a chronic neurodevelopmental disorder that affects up to 3% of the general population. Although epigenetic mechanisms play a role in neurodevelopment disorders, epigenetic pathways associated with OCD have rarely been investigated. Oxytocin is a neuropeptide involved in neurobehavioral functions. Oxytocin has been shown to be associated with the regulation of complex socio-cognitive processes such as attachment, social exploration, and social recognition, as well as anxiety and other stress-related behaviors. Oxytocin has also been linked to the pathophysiology of OCD, albeit inconsistently. The aim of this study was to investigate methylation in two targets sequences located in the exon III of the oxytocin receptor gene (*OXTR*), in OCD patients and healthy controls. We used bisulfite sequencing to quantify DNA methylation in peripheral blood samples collected from 42 OCD patients and 31 healthy controls.

**Results:**

We found that the level of methylation of the cytosine-phosphate-guanine sites in two targets sequences analyzed was greater in the OCD patients than in the controls. The higher methylation in the OCD patients correlated with OCD severity. We measured DNA methylation in the peripheral blood, which prevented us from drawing any conclusions about processes in the central nervous system.

**Conclusion:**

To our knowledge, this is the first study investigating DNA methylation of the *OXTR* in OCD. Further studies are needed to evaluate the roles that DNA methylation and oxytocin play in OCD.

## Background

Obsessive–compulsive disorder (OCD) [1] is a chronic psychiatric illness with a lifetime prevalence of 1–3% in the general population [2]. The results of twin and family studies indicate that inherited factors along with non-shared family environmental factors may be etiologically related to OCD [3]. However, genome wide association studies have failed to find risk alleles of major effect for OCD [4]. Therefore, investigators have expanded the model of susceptibility to include contributions of rare de novo as well as genetic and epigenetic inherited variations [5].

The neuropeptide oxytocin has been linked to emotional regulation processes in recent years. Its therapeutic use has gained momentum, and several trials have investigated the effects of oxytocin administration for the emotional responses of psychiatric disorder patients [6]. Oxytocin anxiolytic properties and its effects over prosocial behaviors have been raised as putative therapeutic characteristics of this molecule. So far, the socializing effects of oxytocin have been shown to be potentially promising for the treatment of autism disorder. In addition, one trial with 15 autistic patients has found that the continuous intravenous administration of oxytocin led to reduction of repetitive behaviors [7].

Paradoxically, while oxytocin is linked to anxiolytic effects and to improvement of repetitive behaviors in autism, elevated oxytocin levels are also putatively involved in the etiology of OCD repetitive behaviors. Clinical trials that investigated the therapeutic use of oxytocin in OCD found no effect of this molecule over the frequency of repetitive symptoms (neither improvement nor worsening) [8, 9]. In contrast, Leckman et al. [10] reported that levels of oxytocin in ventricular cerebrospinal fluid (CSF) are higher in OCD patients than in healthy controls and identified a positive correlation between higher CSF levels of oxytocin and higher frequency of repetitive behaviors. However, in a subsequent study, Altemus et al. [11]. were unable to replicate those findings.

The aforementioned inconsistences in findings relating oxytocin to OCD repetitive behaviors highlight the potential complexity of such association. The absence of effects of the acute administration of oxytocin in neither improvement nor worsening of OCD symptoms suggest that current variation of this neuropeptide may not be relevant for the better understanding of OCD etiology [8, 9]. However, despite the lack of significance of current oxytocin levels, pre-natal and early-natal exposure to high levels of oxytocin could still be potentially related to the future outbreak of OCD repetitive behaviors. Corroborating this hypothesis, oxyctocin has been previously shown to moderate the effects of early social experiences in later life [12].

Epigenetics is the study of the biological mechanisms that explain how environmental influences result in dynamic variations in physiology and adaptive behaviors without changes in the DNA sequence. There are developmental windows of epigenetic reprogramming such that exposures occurring during these dynamic periods are more likely to produce changes to the epigenome [13]. Therefore, the investigation of epigenetic markers might be a tool to understand the effect of events that occurred at critical periods of the development and later along the life spam.

The most widely studied epigenetic alteration in humans is DNA methylation (DNAm), which is one of the main epigenetic mechanisms that control gene expression [14]. In mammalian cells, most DNA methylation occurs on cytosines that precede a guanine nucleotide at loci known as cytosine-phosphate-guanine (CpG) sites [14]. Methylation of oxytocin receptor gene (*OXTR*) has been associated with autism [15], psychopathy [16], acute psychosocial stress in the population experienced war adversities early in life [17] and anorexia [18].

The objective of this study was to assess DNA methylation in the *OXTR* gene in patients with OCD and in healthy controls. We observed two targets sequences located in the exon III of *OXTR* gene, which are part of the CpG island comprising exons I–III [17]. We hypothesized that the OCD patients would show alterations in *OXTR* methylation possibly related to epigenetic reprogramming in critical periods of development.

## Methods

In this study, we evaluated 43 OCD outpatients and 34 healthy controls (i.e., individuals with no history of an Axis I mental disorder). We recruited subjects from the general population through media advertisements, and we conducted all evaluations at the University of São Paulo School of Medicine Institute of Psychiatry, in the city of São Paulo, Brazil, between 2006 and 2008. Patients and healthy subjects also participated in other clinical trials conducted by our group [19, 20]. We applied the following inclusion criteria: being between 18 and 65 years of age; having received a primary DSM-IV diagnosis of OCD; and having a Yale-Brown Obsessive–Compulsive Scale (Y-BOCS) [21] score of ≥16 for obsessions and compulsions or ≥10 for obsessions or compulsions alone. We excluded individuals who had previous history of substance abuse or dependence, psychosis, or head injury with loss of consciousness, as well as those at risk for suicide, or with any medical disorder that could affect the central nervous system, and those who were pregnant. Controls were selected among hospital and university staff. They were selected according to the same criteria described to the patients (except for the presence of OCD) and had no current history of neurological or psychiatric disorders on the basis of SCID interviews. All participants provided written informed consent, which had been approved by the local Institutional Review Board.

In our clinical assessment of the participants, we applied the semi-structured and structured interviews employed in the Brazilian Research Consortium on Obsessive–Compulsive Spectrum Disorders project [22]: the Structured Clinical Interview for DSM-IV Axis I Disorders; those with any Axis I psychiatric diagnosis, and also symptomatology scales as the Beck Depression Inventory (BDI) [23]; the Yale Global Tic Severity Scale [24]; and the Beck Anxiety Inventory (BAI) [25]. We quantified level of education as years of schooling minus the number of grades repeated.

Of the 43 OCD patients evaluated, 33 were treatment-naïve, six had received fluoxetine for 2 weeks, and three had received cognitive-behavior therapy for 2 weeks [26]. Treatment was part of other research protocols taking place at the same institution. Such protocols were not related to this study’s methodology.

### Genomic DNA extraction

We obtained DNA from peripheral blood leucocytes. The blood was drawn before treatment initiation or up to two weeks of undergoing treatment. We subsequently extracted genomic DNA using the “salting-out” method [27, 28].

### DNA methylation analysis

We treated genomic DNA (1000 ng) with sodium bisulfite, using the EpiTect Bisulfite Kit (QIAGEN) in accordance with the manufacturer’s standard protocol. We performed bisulfite-specific polymerase chain reaction (PCR) amplification of two target sequences is located in the exon III of *OXTR* gene (*OXTR1*—cat. PM00016821 and *OXTR2*—cat. PM00016828, QIAGEN), using the PyroMark PCR Kit (QIAGEN). Both target sequences are in the CpG island comprising exons I–III [17]. This CpG island had been already associated with the regulation of *OXTR* gene expression in specific tissue [29].

**Fig. 1:**
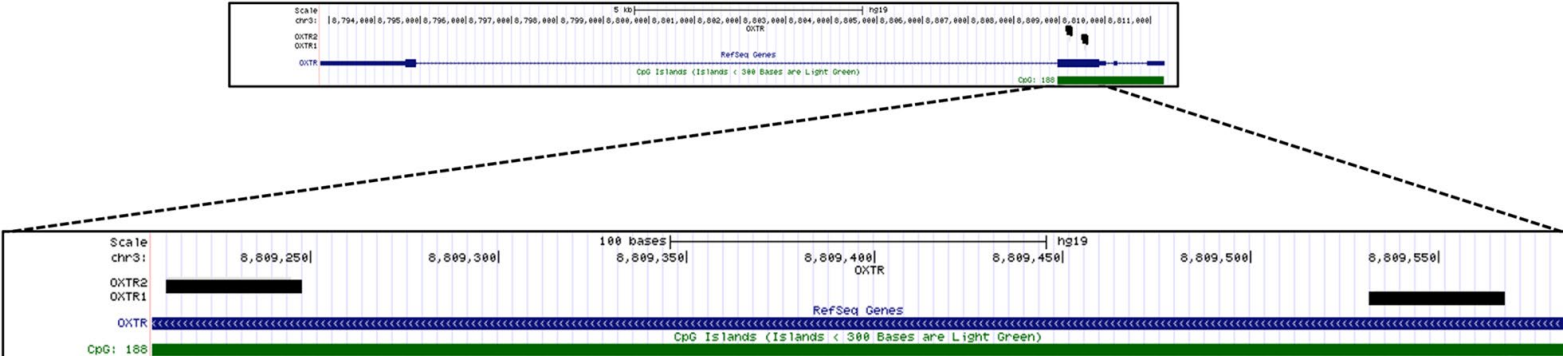
Representation of the two sequence targets in the exon III of the *OXTR* gene. Legend: Representation of *OXTR* gene with the CpG island in *green* and the location of exon III in *blue*. The *dashed lines* show the amplification of the region analyzed that contains two targets, OXTR1 OXTR2. The target sequences are in the exon III of *OXTR* gene (*OXTR1*—cat. PM00016821 and *OXTR2*—cat. PM00016828, QIAGEN), using the PyroMark PCR Kit (QIAGEN). Both regions are in the CpG island the comprise exons I–III. The OXTR1 target sequence has 34 pb with 4 CpG sites analyzed and the OXTR2 target sequence has 34 bp with 5 CpG sites analyzed

The OXTR1 target sequence has 34 pb with 4 CpG sites analyzed (AGGCGGCACAGCAGGTCGGGCCCGTAGAAGCGGA) and the OXTR2 target sequence has 34 bp with 5 CpG sites analyzed (CCGTAGCAGGYAGCGAGCACGATGACCGGCACGA) (Fig. 1).

Using the PyroMark PCR Kit, we generated amplicons in a reaction volume of 25 µl containing 250 nM each of the forward and reverse PCR primers, together with 1 µl (≈50 ng) of bisulfite-treated DNA. We performed the PCR with the following conditions for all reactions: 95 °C for 15 min; 42 cycles at 95 °C for 30 s, 56 °C for 30 s, and 72 °C for 30 s; 1 cycle at 72 °C for 5 min; and a final extension at 4 °C. We prepared single-stranded biotinylated PCR products for sequencing using the Pyrosequencing Vacuum Prep Tool (QIAGEN) in accordance with the manufacturer’s instructions. For each assay, we added 0.3 µM of the sequencing primer in 25 µl of annealing buffer per well. Pyrosequencing is a quantitative sequencing method that allows cytosine methylation to be quantified (in %) at each CpG site within a given sequence. We also applied DNA methylation quality controls, blinding evaluators to the diagnosis and randomly distributing the samples on plates, thus avoiding experimental biases, as well as assessing the quality of the pyrosequencing (including peak height, deviation from the reference sequence pattern, and unexpected peak height).

Previous studies have shown that the Pyrosequencing method is adequate for the evaluation of methylation patterns that are gene-specific [30]. In addition, in comparison with other methodologies, Pyrosequencing has been shown to yield similar results for methylation patterns [31].

We analyzed the data using PyroMark Q24 allele quantification software, version 2.0.6 (QIAGEN), which allowed us to calculate the proportional methylation. Each site received a quality score of “passed”, “check”, or “failed”. The minimum signal value for a peak to achieve a quality score of “passed” in the base-called sequence was 10. The minimum signal value for a peak to achieve a quality score of “check” in the base-called sequence was 5. We assigned a quality score of “failed” to peaks with a signal value lower than 5. We excluded “failed” samples from the analysis, and we reanalyzed samples with peak signal values between 5 and 9. All of the blank controls used in the quality control process tested negative.

### Statistical analysis

To compare the OCD patients and the healthy controls, in terms of the common symptomatology (BDI and BAI scores) and demographic data, we used nonparametric method for comparing medians between populations, named *Quor*, as proposed by [32]. This method calculate the confidence statement that the median of patients population is less than the median of controls population or (vice versa) [32, 33]. We tested the normal distribution of data related to the *OXTR* CpG sites, using the Shapiro–Wilk test. The data of CpG sites related to the *OXTR1 and OXTR2* target sequence did not present normal distribution for patients and controls. Therefore, in subsequent analyses, we employed tests that do not assume normal distribution. To identify differences in methylation sites between patients and controls, we used Hotelling’s *T* ^*2*^ test with Chi-square approximation, [34] which is a multivariate test, analog of the univariate two-sample *t* test, to compare the means of two multivariate population. We conducted post hoc analyses using the *Quor* method [35]. After the post hoc analysis, we used Kendall’s tau rank correlation coefficient test to determine whether the *OXTR* CpG sites in both target sequences were associated with clinical and demographic variables (age, sex, level of education, BDI score, BAI score, tics, and Y-BOCS score). Kendall’s tau rank correlation coefficient test is a nonparametric rank correlation test for hypothesis testing in small samples for which the data present non-normal distributions [36].

We performed a linear regression analysis for the log of the mean of methylation levels considering both targets sequences for CpG *OXTR* gene, including clinical and demographic variables (age, sex, level of education, BDI score, BAI score, tics, and Y-BOCS score). The level of significance was set at *p* ≤ 0.05. All statistical tests were two-tailed, and we performed all statistical analyses using the software R, version 2.14.1 (R Development Core Team).

## Results

After the pyrosequencing analysis, one patient and three controls were excluded by lower quality of score to analyze the methylation. Therefore, the final sample comprised 42 OCD patients and 31 healthy controls. Table 1 shows the clinical and demographic characteristics of the participants. The median age, sex, and level of education was similar between the two groups. The OCD patients scored higher on the BDI and BAI, indicating that the population median for the healthy controls was lower than was that of the OCD patients, with a high confidence.

**Table 1:** Demographic and clinical characteristics of patients with obsessive–compulsive disorder and healthy controls

Characteristic	OCD patients	Healthy controls	*p*	Confidence^a^	
	(*n* = 42)	(*n* = 31)		MpC < MpP	MpP < MpC
Age	29.38/29 (8.65)	28.55/28 (9.28)	–	0.28	0.28
Sex (male)	61%	64%	0.98	–	–
Years of schooling	13.67/13 (3.07)	15.71/15 (4.4)	–	0.01	0.58
BDI	18.69/20 (8.86)	2.13/1 (2.38)	–	1.00	0.00
BAI	18.33/18 (11.1)	1.71/1 (1.81)	–	1.00	0.00

Except for sex, values are presented as mean/median (SD)

*OCD* obsessive–compulsive disorder, *BDI* Beck Depression Inventory, *BAI* Beck Anxiety Inventory, *MpC* population median for the control group, *MpP* population median for the (OCD) patient group

^a^We used confidences (determined via the Quor method) in order to compare the population medians between the patient and control groups

### Methylation patterns

**Fig. 2:**
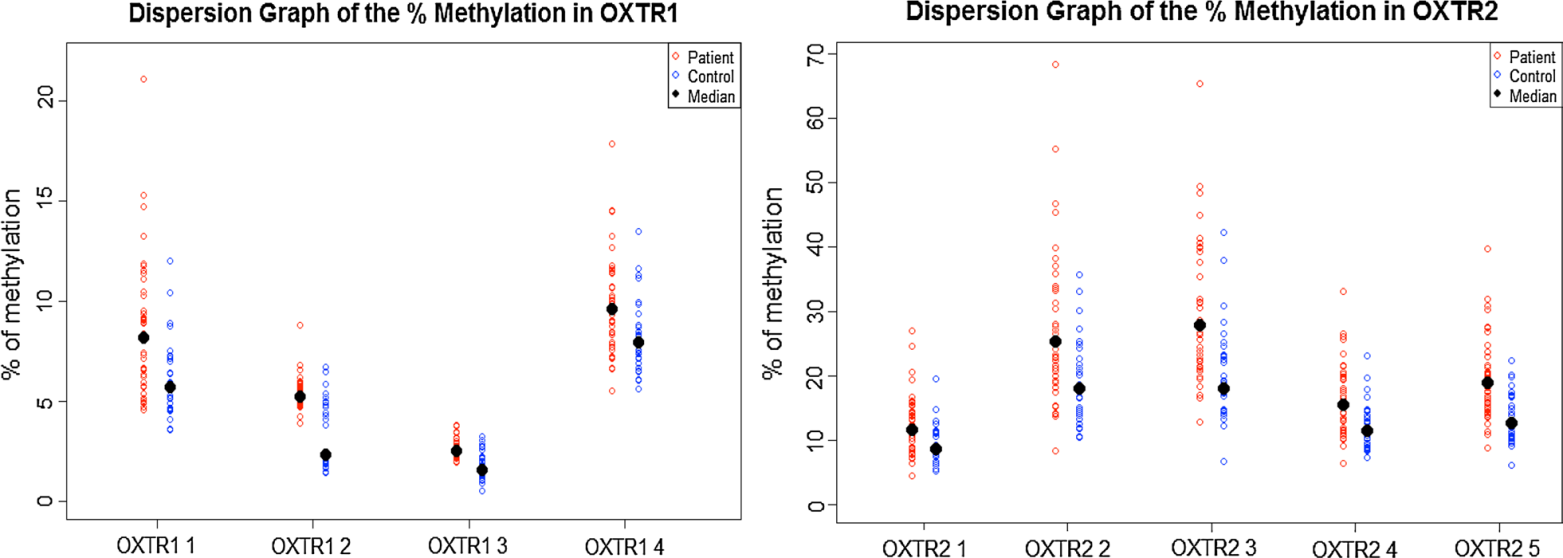
Dispersion graph of the % of methylation in OXTR1 and OXTR2. Legend: *Red* and *blue dots* represent the % of methylation in the two target sequences in the exon III of *OXTR* gene in OCD individuals and controls, respectively. The *black dot* represents the median

The dispersion graph displayed in Fig. 2 shows the proportional methylation in the two target sequences located in the exon III of *OXTR* gene in OCD patients and healthy controls. The two target sequences evaluated (one with 5 and other with 4 CpG sites) had levels of methylation that differed significantly between OCD patients and healthy controls (*OXTR1*—*T* ^*2*^ = 59.2; *p* < 0.001; and *OXTR2*—*T* ^*2*^ = 26.6; *p* < 0.001). We also performed the Hotelling’s *T* ^*2*^ test with the Chi square approximation between the controls and patients who received medication or CBT and drug naïve. The level of methylation did not differ between the drug-naïve patients (n = 33) and the patients who had already started any treatment (n = 9). Although is important to note that the sample size is rather low to support an analysis of sub-group effects.

Post-hoc analysis showed high confidence (>0.9) for a statement that the population median for all CpG sites of the *OXTR* 1 and OXTR 2 target sequences were lower in the healthy control group than in the OCD patients group (Table 2).

**Table 2:** Median methylation levels of cytosine-phosphate-guanine units in patients with obsessive–compulsive disorder and healthy controls

*OXTR* gene	CpG island unit	OCD patients	Healthy controls	Confidence
		(*n* = 42)	(*n* = 31)	
		Median	Median	MpC < MpP
*OXTR1*	1	8.19	5.71	0.9608
*OXTR1*	2	5.24	2.32	0.9995
*OXTR1*	3	2.53	1.55	0.9983
*OXTR1*	4	9.62	7.97	0.9436
*OXTR2*	1	11.65	8.62	0.9275
*OXTR2*	2	25.46	18.01	0.9591
*OXTR2*	3	27.86	19.09	0.9846
*OXTR2*	4	15.50	11.50	0.9559
*OXTR2*	5	18.99	12.65	0.9625

*OCD* obsessive–compulsive disorder, *CpG* cytosine-phosphate-guanine; *OXTR* oxytocin receptor, *MpC* population median for the control group, *MpP* population median for the (OCD) patient group

To determine whether any of the nine CpG sites correlated with specific clinical features that might be related with methylation, we observed if BDI score, BAI score, positive diagnosis of tic disorder, and the severity of OCD symptoms, as well as with age, sex, and level of education, in the OCD patients were correlate with any result of methylation in CpG sites (Table 3). In some of the *OXTR1* CpG sites (CpG units 1, 3, and 4) and in all of the *OXTR2* CpG sites, the individual methylation level correlated negatively with the BDI score (Table 3). Although three of the OCD patients had been diagnosed with a tic disorder, we found no correlation between tics and the level of methylation.

**Table 3:** Kendall’s correlation analysis of methylation status of the oxytocin receptor gene in relation to demographic and clinical variables in patients with obsessive–compulsive disorder (*n* = 42)

*OXTR* gene	CpG island unit	Age	Sex	Level of education	BDI	BAI	Tics	Y-BOCS
*OXTR1*	1	τ = 0.046 (0.679)	τ = 0.001 (0.999)	τ = −0.089 (0.434)	τ = −0.272 (0.013)	τ = −0.115 (0.296)	τ = −0.082 (0.534)	τ = 0.013 (0.913)
*OXTR1*	2	τ = 0.034 (0.760)	τ = 0.157 (0.227)	τ = −0.029 (0.799)	τ = −0.141 (0.199)	τ = −0.055 (0.617)	τ = 0.082 (0.534)	τ = 0.058 (0.601)
*OXTR1*	3	τ = 0.086 (0.434)	τ = 0.168 (0.198)	τ = 0.059 (0.604)	τ = −0.322 (0.003)	τ = −0.122 (0.267)	τ = 0.087 (0.509)	τ = −0.118 (0.286)
*OXTR1*	4	τ = 0.150 (0.171)	τ = 0.011 (0.937)	τ = −0.009 (0.938)	τ = −0.336 (0.002)	τ = −0.167 (0.128)	τ = −0.077 (0.560)	τ = −0.029 (0.793)
*OXTR2*	1	τ = −0.023 (0.839)	τ = −0.119 (0.371)	τ = −0.074 (0.522)	τ = −0.284 (0.010)	τ = −0.165 (0.137)	τ = −0.156 (0.239)	τ = −0.033 (0.769)
*OXTR2*	2	τ = −0.023 (0.839)	τ = −0.129 (0.329)	τ = −0.053 (0.647)	τ = −0.244 (0.028)	τ = −0.153 (0.169)	τ = −0.135 (0.309)	τ = −0.041 (0.718)
*OXTR2*	3	τ = 0.026 (0.821)	τ = −0.039 (0.772)	τ = −0.069 (0.552)	τ = −0.264 (0.017)	τ = −0.138 (0.215)	τ = −0.098 (0.461)	τ = −0.043 (0.701)
*OXTR2*	4	τ = −0.021 (0.857)	τ = −0.068 (0.611)	τ = −0.050 (0.663)	τ = −0.234 (0.035)	τ = −0.130 (0.241)	τ = −0.104 (0.437)	τ = −0.046 (0.684)
*OXTR2*	5	τ = −0.021 (0.857)	τ = −0.071 (0.591)	τ = −0.066 (0.567)	τ = −0.259 (0.019)	τ = −0.135 (0.223)	τ = −0.072 (0.590)	τ = −0.048 (0.668)

*OXTR* oxytocin receptor, *CpG* cytosine-phosphate-guanine, *BDI* Beck Depression Scale Inventory, *BAI* Beck Anxiety Scale Inventory, *Y-BOCS* Yale-Brown Obsessive–Compulsive Scale, *τ* Kendall’s tau rank correlation coefficient

Values are presented as τ (*p*)

After that we performed a linear regression analysis, using log of the mean of methylation levels considering both targets sequences for CpG *OXTR* gene and the clinical and demographic variables as a predictor of level of methylation (age, sex, level of education, BDI score, BAI score, tics, and Y-BOCS score). We found that the Y-BOCS score was the main determinant of the methylation level (β = 0.02; *p* < 0.001) and also the BDI score (β = − 0.013; *p* = 0.0037) (Table 4).

**Table 4:** Regression model for the log of the mean of methylation levels of the oxytocin receptor 1 gene in patients with obsessive–compulsive disorder

Parameter	Estimate	SE	95% CI	*p*
Intercept	2.34	0.057	2.22–2.45	<0.001
BDI	−0.013	0.006	−0.025 to −0.0014	0.029
Y-BOCS	0.02	0.005	0.01–0.03	<0.001

*BDI* Beck Depression Scale Inventory, *Y-BOCS* Yale-Brown Obsessive–Compulsive Scale

## Discussion

To our knowledge, this is the first study investigating *OXTR* DNA methylation in OCD. Our analysis revealed hypermethylation in the CpG sites of two sequences targets located in the exon III, which are part of the CpG island that comprise exons I–III of *OXTR* gene, in patients with OCD when compared to healthy subjects and a positive correlation between hypermethylation and OCD symptom severity. A number of studies have shown that this CpG island that comprise exons I–III, is associated with transcription of the *OXTR* gene [17, 29, 37, 38]. Therefore, the high levels of DNA methylation observed in both of the *OXTR* sequences targeted in our study could be related with the transcription of the *OXTR* gene.

In a previous study, Gregory et al. investigated the CpG island that overlaps exons I–III of *OXTR* gene in a cohort of autistic patients and healthy subjects. When compared to healthy subjects, autistic patients had hypermethylated *OXTR* in two different types of tissue: the peripheral blood mononuclear cells and in the temporal cortex. The similarities in epigenetic profiles from these two tissue types of ectodermal (peripheral blood) and mesodermal (temporal cortex) origin are possible since epigenetic profiles undergo reprogramming during very early gestation [15]. Therefore, our finding might indicate that, at some critical point of development, environmental factors led to hypermethylation of the *OXTR* in OCD patients but not in healthy subjects.

In rats, grooming behaviors are a putative model for OCD, and the injection of oxytocin into the amygdala has been shown to induce grooming behaviors in those animals [39]. Since oxytocin increases grooming and, in other circumstances, reduces fear, it is tempting to hypothesize that oxytocin-induced grooming behaviors are anxiolytic. In OCD, compulsions are performed to alleviate anxiety corroborating the idea of the anxiolytic effect of grooming [40]. Therefore, *OXTR* hypermethylation could be a marker of higher oxytocin levels during a critical period of development when “grooming-like behaviors” are established as “preferential counter-anxiety behaviors”. It is important to highlight, however, that this hypothesis remains highly speculative at this point.

In the present study, we also found a negative correlation between the level of methylation and the severity of depressive symptoms. Previously, higher CSF levels of oxytocin had been associated with higher severity of depressive symptoms in children and adolescents with OCD [41]. As well, a positive correlation between baseline plasma and salivary oxytocin levels and depressive symptoms has been described in healthy subjects [42].

However, in patients with major depression, lower concentrations of cerebrospinal fluid and plasmatic levels of oxytocin have been associated with lower severity of depressive symptoms [43]. Such inconsistences suggest that, similarly to what occurs with OCD, the relationship of oxytocin and depressive symptoms might not be related to current oxytocin levels. More likely, previous exposure to environmental factors that led to *OXTR* hypermethylation might impact the likelihood of later emergence of depressive symptoms.

The interpretation of our results should to be regarded with caution due to the gaps in our current knowledge about the mechanisms of epigenetic control along the life cycle. Although environmental influences and stochastic events can cause changes in the pattern of DNA methylation, these epigenetic markers are mostly susceptible to change during epigenetic reprogramming that occurs preferentially but not exclusively in early development [44–46]. In addition, epigenetic markers are tissue-specific and site-specific. Therefore, the methylation pattern found in a specific population of one cell type does not necessarily reflect the methylation pattern of other cell types, such as neurons. In our study, we did not confirm if peripheral patterns of methylation reflected the patterns of other tissue types. Therefore, we cannot determine in which tissue types the hypermethylation of the *OXTR* was established in our sample. We also do not have information about the current oxytocin levels in blood of OCD patients and controls and could not evaluate if hypermethylation of OXTR had any impact on peripheral availability of the neuropeptide oxytocin.

## Conclusion

Our findings suggest that, at some critical point of development, environmental factors led to hypermethylation of the *OXTR* in OCD patients but not in healthy subjects. Future studies should emphasize the gene-environmental relationships that might explain the hypermethylation of the *OXTR* in the pre-natal period and later emergence of OCD.

## Abbreviations

OCD: obsessive–compulsive disorder
CSF: ventricular cerebrospinal fluid
DNAm: DNA methylation
CpG: cytosine-phosphate-guanine
*OXTR*: oxytocin receptor gene
Y-BOCS: Yale-Brown Obsessive–Compulsive Scale
BDI: Beck Depression Inventory
BAI: Beck Anxiety Inventory
PCR: polymerase chain reaction

## References

[1] Atli A, Boysan M, Çetinkaya N, Bulut M, Bez Y (2014). Latent class analysis of obsessive–compulsive symptoms in a clinical sample. Compr Psychiatry.

[2] Ruscio AM, Stein DJ, Chiu WT, Kessler RC (2010). The epidemiology of obsessive–compulsive disorder in the National Comorbidity Survey Replication. Mol Psychiatry.

[3] Pauls DL, Abramovitch A, Rauch SL, Geller DA (2014). Obsessive–compulsive disorder: an integrative genetic and neurobiological perspective. Nat Rev Neurosci.

[4] Stewart SE, Yu D, Scharf JM, Neale BM, Fagerness JA, Mathews CA, Arnold PD, Evans PD, Gamazon ER, Davis LK (2013). Genome-wide association study of obsessive–compulsive disorder. Mol Psychiatry.

[5] Malhotra D, Sebat J (2012). CNVs: harbingers of a rare variant revolution in psychiatric genetics. Cell.

[6] Cochran DM, Fallon D, Hill M, Frazier JA (2013). The role of oxytocin in psychiatric disorders: a review of biological and therapeutic research findings. Harv Rev Psychiatry.

[7] Hollander E, Novotny S, Hanratty M, Yaffe R, DeCaria CM, Aronowitz BR, Mosovich S (2003). Oxytocin infusion reduces repetitive behaviors in adults with autistic and Asperger’s disorders. Neuropsychopharmacology.

[8] den Boer JA, Westenberg HG (1992). Oxytocin in obsessive compulsive disorder. Peptides.

[9] Epperson CN, McDougle CJ, Price LH (1996). Intranasal oxytocin in obsessive–compulsive disorder. Biol Psychiatry.

[10] Leckman JF, Goodman WK, North WG, Chappell PB, Price LH, Pauls DL, Anderson GM, Riddle MA, McSwiggan-Hardin M, McDougle CJ (1994). Elevated cerebrospinal fluid levels of oxytocin in obsessive–compulsive disorder. Comparison with Tourette’s syndrome and healthy controls. Arch Gen Psychiatry.

[11] Altemus M, Jacobson KR, Debellis M, Kling M, Pigott T, Murphy DL, Gold PW (1999). Normal CSF oxytocin and NPY levels in OCD. Biol Psychiatry.

[12] Cushing BS, Kramer KM (2005). Mechanisms underlying epigenetic effects of early social experience: the role of neuropeptides and steroids. Neurosci Biobehav Rev.

[13] Bale TL (2015). Epigenetic and transgenerational reprogramming of brain development. Nat Rev Neurosci.

[14] Kumsta R, Hummel E, Chen FS, Heinrichs M (2013). Epigenetic regulation of the oxytocin receptor gene: implications for behavioral neuroscience. Front Neurosci.

[15] Gregory SG, Connelly JJ, Towers AJ, Johnson J, Biscocho D, Markunas CA, Lintas C, Abramson RK, Wright HH, Ellis P (2009). Genomic and epigenetic evidence for oxytocin receptor deficiency in autism. BMC Med.

[16] Dadds MR, Moul C, Cauchi A, Dobson-Stone C, Hawes DJ, Brennan J, Ebstein RE (2014). Methylation of the oxytocin receptor gene and oxytocin blood levels in the development of psychopathy. Dev Psychopathol.

[17] Unternaehrer E, Luers P, Mill J, Dempster E, Meyer AH, Staehli S, Lieb R, Hellhammer DH, Meinlschmidt G (2012). Dynamic changes in DNA methylation of stress-associated genes (OXTR, BDNF) after acute psychosocial stress. Transl Psychiatry.

[18] Kim YR, Kim JH, Kim MJ, Treasure J (2014). Differential methylation of the oxytocin receptor gene in patients with anorexia nervosa: a pilot study. PLoS ONE.

[19] Hoexter MQ, de Souza Duran FL, D’Alcante CC, Dougherty DD, Shavitt RG, Lopes AC, Diniz JB, Deckersbach T, Batistuzzo MC, Bressan RA (2012). Gray matter volumes in obsessive–compulsive disorder before and after fluoxetine or cognitive-behavior therapy: a randomized clinical trial. Neuropsychopharmacology.

[20] Hoexter MQ, Shavitt RG, D’Alcante CC, Cecconi JP, Diniz JB, Belotto-Silva C, Hounie AG, Borcato S, Moraes I, Joaquim MA (2009). The drug-naïve OCD patients imaging genetics, cognitive and treatment response study: methods and sample description. Rev Bras Psiquiatr.

[21] Goodman WK, Price LH, Rasmussen SA, Mazure C, Delgado P, Heninger GR, Charney DS (1989). The Yale-Brown Obsessive Compulsive Scale. II. Validity. Arch Gen Psychiatry.

[22] Miguel EC, Ferrão YA, Rosário MC, Mathis MA, Torres AR, Fontenelle LF, Hounie AG, Shavitt RG, Cordioli AV, Gonzalez CH (2008). The Brazilian research consortium on obsessive–compulsive spectrum disorders: recruitment, assessment instruments, methods for the development of multicenter collaborative studies and preliminary results. Rev Bras Psiquiatr.

[23] Beck AT, Beamesderfer A (1974). Assessment of depression: the depression inventory. Mod Probl Pharmacopsychiatry.

[24] Leckman JF, Riddle MA, Hardin MT, Ort SI, Swartz KL, Stevenson J, Cohen DJ (1989). The Yale Global Tic Severity Scale: initial testing of a clinician-rated scale of tic severity. J Am Acad Child Adolesc Psychiatry.

[25] Beck AT, Epstein N, Brown G, Steer RA (1988). An inventory for measuring clinical anxiety: psychometric properties. J Consult Clin Psychol.

[26] Hoexter MQ, Dougherty DD, Shavitt RG, D’Alcante CC, Duran FL, Lopes AC, Diniz JB, Batistuzzo MC, Evans KC, Bressan RA (2013). Differential prefrontal gray matter correlates of treatment response to fluoxetine or cognitive-behavioral therapy in obsessive–compulsive disorder. Eur Neuropsychopharmacol.

[27] Miller SA, Dykes DD, Polesky HF (1988). A simple salting out procedure for extracting DNA from human nucleated cells. Nucleic Acids Res.

[28] Marioni RE, Shah S, McRae AF, Chen BH, Colicino E, Harris SE, Gibson J, Henders AK, Redmond P, Cox SR (2015). DNA methylation age of blood predicts all-cause mortality in later life. Genome Biol.

[29] Kusui C, Kimura T, Ogita K, Nakamura H, Matsumura Y, Koyama M, Azuma C, Murata Y (2001). DNA methylation of the human oxytocin receptor gene promoter regulates tissue-specific gene suppression. Biochem Biophys Res Commun.

[30] Shen L, Kondo Y, Guo Y, Zhang J, Zhang L, Ahmed S, Shu J, Chen X, Waterland RA, Issa JP (2007). Genome-wide profiling of DNA methylation reveals a class of normally methylated CpG island promoters. PLoS Genet.

[31] Claus R, Wilop S, Hielscher T, Sonnet M, Dahl E, Galm O, Jost E, Plass C (2012). A systematic comparison of quantitative high-resolution DNA methylation analysis and methylation-specific PCR. Epigenetics.

[32] Pereira CAdB, Polpo A, MedOr: order of medians based on confidence statements. arXiv preprint arXiv:12125405. 2012.

[33] Pereira CAdB, de Campos CP, Polpo A. Confidence statements for ordering quantiles. arXiv e-prints. 2012.

[34] Tang MH, Varadan V, Kamalakaran S, Zhang MQ, Dimitrova N, Hicks J (2012). Major chromosomal breakpoint intervals in breast cancer co-localize with differentially methylated regions. Front Oncol.

[35] Polpo A, Pereira CAdB. R Package: MedOr. 2012. Available on: https://cran.r-project.org/web/packages/MedOr/vignettes/MedOr.pdf.

[36] Rosner B, Glynn RJ (2007). Interval estimation for rank correlation coefficients based on the probit transformation with extension to measurement error correction of correlated ranked data. Stat Med.

[37] Mamrut S, Harony H, Sood R, Shahar-Gold H, Gainer H, Shi YJ, Barki-Harrington L, Wagner S (2013). DNA methylation of specific CpG sites in the promoter region regulates the transcription of the mouse oxytocin receptor. PLoS ONE.

[38] Harony-Nicolas H, Mamrut S, Brodsky L, Shahar-Gold H, Barki-Harrington L, Wagner S (2014). Brain region-specific methylation in the promoter of the murine oxytocin receptor gene is involved in its expression regulation. Psychoneuroendocrinology.

[39] Marroni SS, Nakano FN, Gati CD, Oliveira JA, Antunes-Rodrigues J, Garcia-Cairasco N (2007). Neuroanatomical and cellular substrates of hypergrooming induced by microinjection of oxytocin in central nucleus of amygdala, an experimental model of compulsive behavior. Mol Psychiatry.

[40] Shavitt RG, de Mathis MA, Oki F, Ferrao YA, Fontenelle LF, Torres AR, Diniz JB, Costa DL, do Rosário MC, Hoexter MQ (2014). Phenomenology of OCD: lessons from a large multicenter study and implications for ICD-11. J Psychiatr Res..

[41] Swedo SE, Leonard HL, Kruesi MJ, Rettew DC, Listwak SJ, Berrettini W, Stipetic M, Hamburger S, Gold PW, Potter WZ (1992). Cerebrospinal fluid neurochemistry in children and adolescents with obsessive–compulsive disorder. Arch Gen Psychiatry.

[42] Holt-Lunstad J, Birmingham W, Light KC (2011). The influence of depressive symptomatology and perceived stress on plasma and salivary oxytocin before, during and after a support enhancement intervention. Psychoneuroendocrinology.

[43] Scantamburlo G, Hansenne M, Fuchs S, Pitchot W, Maréchal P, Pequeux C, Ansseau M, Legros JJ (2007). Plasma oxytocin levels and anxiety in patients with major depression. Psychoneuroendocrinology.

[44] Jaenisch R, Bird A (2003). Epigenetic regulation of gene expression: how the genome integrates intrinsic and environmental signals. Nat Genet.

[45] Heijmans BT, Tobi EW, Lumey LH, Slagboom PE (2009). The epigenome: archive of the prenatal environment. Epigenetics.

[46] Heijmans BT, Mill J (2012). Commentary: the seven plagues of epigenetic epidemiology. Int J Epidemiol.

